# Development of the children's primitive reflex integration assessment scale

**DOI:** 10.3389/fpsyg.2025.1495990

**Published:** 2025-01-22

**Authors:** Meng Wang, Jing Yu, Hongyao Li, Chongran Zhao, Yichao Li, Xinyue Yang

**Affiliations:** ^1^College of Sports Science, Shenyang Normal University, Shenyang, China; ^2^Martial Arts and Dance Academy, Shenyang Sport University, Shenyang, China; ^3^Department of Physical Education, Keimyung University, Daegu, Republic of Korea

**Keywords:** primitive reflexes, children, motor function disorder, cognitive psychology, neurodevelopmental disorder

## Abstract

**Objective:**

Non-integrated primitive reflexes (PRs) in children can lead to issues in motor function and psychological wellbeing, while prior studies have shown correlations between PR integration and neurodevelopmental disorders in children. However, measurement methods for PR integration remain unestablished. Therefore, in the present study, we describe the development of a measurement scale for PR integration, a novel assessment tool to evaluate PR integration in children.

**Methods:**

Combining a literature review, practical experience, and results of specialized group discussions, a preliminary draft of the Children's Primitive Reflex Integration Measurement Scale (CPRIMS) was formulated. Employing a convenience sampling method, participants were selected from first and second-grade students in three primary schools in Liaoning province, Shenyang city, from May to July 2023. Item Discrimination Method (IDM), Critical Ratio Method (CRM), and Internal Consistency Coefficient Method (ICCM) were used for item analysis of pilot testing data. For formal testing data, Cronbach's α assessed the reliability of the scale, while fit indices such as chi-square value/degrees of freedom (χ^2^/df), Root Mean Square Error of Approximation (RMSEA), and Comparative Fit Index (CFI), along with tests of construct validity, evaluated the scale's validity.

**Results:**

Overall, 555 participants were selected, 234 children with a mean age of 7.59 ± 0.71 years participated in the pilot testing, while 321 children with a mean age of 7.73 ± 0.71 years participated in the formal testing. CPRIMS comprises seven dimensions and seventeen items, including the Moro reflex (MR), Asymmetrical Tonic Neck Reflex (ATNR), Symmetrical Tonic Neck Reflex (STNR), Tonic Labyrinthine Reflex (TLR), Spinal Galant Reflex (SGR), Spinal Perez Reflex (SPR), and Landau Reflex (LR), explaining 88.2% of the total variance. Confirmatory factor analysis revealed a good model fit (χ^2^/df = 1.631, RMSEA = 0.044, NFI = 0.950, CFI = 0.980, IFI = 0.932, TLI = 0.972). Cronbach's α coefficients for the seven dimensions ranged from 0.730 to 0.945, demonstrating strong reliability.

**Conclusion:**

CPRIMS, which includes dimensions such as MR, ATNR, STNR, TLR, SGR, SPR, and LR, demonstrates strong reliability and validity, indicating that this measure could serve as a reliable and effective tool for assessing the integration levels of PRs in children aged 6 to 9 years old.

## 1 Introduction

Primitive reflexes (PRs) develop during the fetal or infant stages. During infancy, more than 20 types of PRs are exhibited, including swallowing, breathing, visual and auditory responses, head movements, hand grasping movements, trunk control, and lower limb movements, among others (Bilbilaj et al., 2017). As infants grow and develop, most PRs gradually integrate and evolve into more complex patterns, enabling self-control of body movements and motor skills (Berne, 2006); however, a small portion of these reflexes persist (Wang et al., 2023). PRs initially serve to protect newborns from external stimuli and are gradually integrated into purposeful movements (Berne, 2006). However, if PRs fail to integrate within an appropriate timeframe, they may impact the brain's ability to effectively process sensory information (Blythe, 1996) and lead to motor dysfunction in children (Adams and Craft, 2014), as well as cognitive and psychological issues, and neurodevelopmental disorders (Wang et al., 2023). Specifically, compared to children with non-integrated PRs, those with non-integrated PRs can experience motor dysfunctions (Adams and Craft, 2014), such as difficulties in handling fine and gross motor tasks, poor balance, and challenges with hand-eye coordination (Wang et al., 2023; Blythe, 1996; Adams and Craft, 2014; Blythe, 2002). In terms of cognitive and psychological aspects, children with non-integrated PRs may further struggle with interpersonal interactions, exhibit higher levels of anxiety and oppositional behavior (Melillo et al., 2020; Quevedo et al., 2015; Pecuch et al., 2020; Yang, 2024), and could display hyperactive or impulsive behaviors, particularly during cognitively demanding tasks, or when required to maintain focus for extended periods, which can lead to learning difficulties (Taylor et al., 2004; Konicarova et al., 2013). Additionally, non-integrated PRs have been associated with neurodevelopmental disorders, such as bipolar disorder (Tuysuzoglu et al., 2011), cerebral palsy with movement disorders (Kyllerman et al., 1982; Eek et al., 2018), and dyslexia (Chinello et al., 2018), and are also associated with learning disabilities (Hermann, 2012; Grzywniak, 2017; Feldhacker et al., 2021), scoliosis (Gieysztor et al., 2018), and eating issues (Gieysztor et al., 2022; Hobo et al., 2014).

These challenges can have profound negative impacts on a child's life. As such, the assessment of PR integration holds significant value in the treatment and prevention of motor dysfunctions, mental health issues, and neurodevelopmental disorders in children. However, current research and attention in the field of PRs remain insufficient. Through a review of the literature, it was found that existing measurement tools are not yet well-developed. The INPP Reflex Assessment Test (Blythe, 2017) was adapted from the original Blythe-McGlown Child Questionnaire, initially designed by Blythe and McGlown in 1979, revised in 1998, and further modified by Goddard Blythe in 2006. Nevertheless, the questionnaire heavily relies on comprehensive information, including detailed developmental data from pregnancy to school age. Collecting such data demands respondents to possess strong memory and precise expression skills, which significantly increases the difficulty of data collection. The Bender-Purdue Reflex Test (Bender, 1978) relies on parents or teachers to judge whether a child's PRs are non-integrated based on behavioral and psychological traits associated with different types of children. However, this questionnaire emphasizes superficial behavioral phenomena (e.g., “Not really hyperkinetic or immature” and “Easily distracted by anything and everything”) rather than directly assessing children's primitive reflex integration through specific physical actions, potentially affecting the accuracy of evaluation results. The Schilder Test and Goddard Test (Morrison, 1985; Hickey and Feldhacker, 2022) include well-structured measurement procedures, observation methods, and scoring standards. But they were developed in 2005 and lack clear validity and reliability indicators. Functionally, PRs primarily assist newborns and infants in interacting with their environment through reflexive actions before cortical control is established (Hickey and Feldhacker, 2022). Yet, given the rapid evolution of society, questions arise as to whether the types of non-integrated PRs have changed, whether such phenomena have become more prevalent among children, or whether new characteristics have emerged. Furthermore, inconsistencies in the description of scoring standards for these measurement methods warrant further exploration.

Additionally, our review of studies employing the Schilder Test and Goddard Test revealed variability in the age of participants: Konicarova and Bob (2012) assessed children aged 8–11 years, while Hickey and Feldhacker (2022) focused on children aged 4–6 years. Retained Symmetric Tonic Neck Reflex (STNR), Asymmetric Tonic Neck Reflex (ATNR), and Moro Reflex (MR) can result in behavioral and psychological issues such as anxiety, reading difficulties, and time management problems (Taylor et al., 2004; Gieysztor et al., 2018; Hickey and Feldhacker, 2022). These issues often go undetected before school age but become more apparent as children approach or enter school. To further investigate the behavioral and psychological problems caused by non-integrated PRs, this study aims to explore the integration of PRs in children aged 6–9 years. The study will pioneer the development of the Children's Primitive Reflex Integration Measurement Scale (CPRIMS) to provide a reliable tool for scientifically and objectively assessing the integration levels of PRs in children.

## 2 Participants and methods

### 2.1 Participant screening

To construct the sample, a convenience sampling method was applied during May–June 2023 to collect first and second-grade students from three primary schools in Shenyang, China as participants for the pilot study. In June–July 2023, the same method was applied to select students from the same three schools as participants for the formal study. The sample size for the item selection and validity-reliability analysis were determined based on the standard suggested by prior research (Kline, 2011), which recommends a ratio of 5 to 10 participants per item.

The inclusion criteria were as follows: (1) Agreed to voluntarily participate in the measurement; (2) Normal or corrected to normal hearing and vision; and (3) Normal intellectual level. The exclusion criteria were as follows: (1) Lack of basic comprehension and communication abilities; (2) Prior participation in similar types of measurements; (3) Any physical illnesses. Following the application of these criteria, 555 students participated in this study. All participants were recruited through their schools, and written consent was provided by parents or legal guardians. This study was approved by the Ethics Committee of Shenyang Sport University, Liaoning Province, China.

### 2.2 Initial items for scale development

The item pool and structure of the Primitive Reflex Integration Measurement Scale were developed as follows: First, the methods for evaluating Primitive Reflex Integration in previous studies (Wagner, 2015; Geerlinks, 2018; Feldhacker et al., 2022; Futagi et al., 2012; Bob et al., 2021; Futagi et al., 1992; Zafeiriou et al., 1995, 1999a,b; Zafeiriou, 2000; Konicarova and Bob, 2012, 2013) were organized and combined with practical experience. A panel discussion was then held, involving five special education teachers and five pediatricians, to review, analyze, and refine each item. Adjustments were made to the categorization of dimensions and the simplification of expressions. Initially, the draft scale consisted of 45 items across 13 dimensions, which was then refined to 37 items across 12 dimensions to create the Primitive Reflex Integration Pilot Scale. The final categorization of dimensions would be determined through confirmatory factor analysis. The scale's framework includes the following reflexes: MR, ATNR, STNR, Tonic Labyrinthine Reflex (TLR), Spinal Galant Reflex (SGR), Spinal Perez Reflex (SPR), Landau Reflex (LR), Palmar Grasp Reflex (PAGR), Plantar Grasp Reflex (PLGR), Babkin Reflex (BNR), Hands Pulling Reflex (HPR), and Babinski Reflex (BIR). A 4-point Likert scale was used (0 = Not Present, 1 = Slight, 2 = Noticeable, 3 = Very Noticeable) to form the scoring criteria for the pilot scale (Xue et al., 2023). The measurement was conducted by 10 trained researchers in the relevant field, working in pairs to independently observe and score each participant. If there was a discrepancy in scoring, a third researcher was brought in to resolve the inconsistency.

## 3 Statistical methods

### 3.1 Item analysis

Pilot data were analyzed using IBM SPSS Statistics 26.0, employing the following item analysis methods: (1) Item Discrimination Method (IDM): Participants were ranked based on their total scores, with the top 27% categorized as the high-score group, and the bottom 27% as the low-score group. The item discrimination value for each item was calculated by dividing the difference between the mean scores of the high and low groups by the range of scores. Items with a discrimination value of <0.2 were removed (Ebel and Frisbie, 1991); (2) Critical Ratio Method: An independent sample *t-*test was applied to compare the mean scores between the high and low groups. Items were deleted if the difference did not reach statistical significance (*P* > 0.05), or if the critical ratio was <3 (Wu, 2010); (3) Internal Consistency Coefficient Method (ICCM): The Cronbach's α coefficient was calculated following the sequential deletion of each item. If the resulting Cronbach's α was greater than the Cronbach's α of the overall scale, the item was considered to reduce the internal consistency of the scale, and was deleted (Lance et al., 2006).

### 3.2 Reliability and validity analysis

Reliability and validity analysis were conducted using the formal measurement data. The reliability of the scale and its dimensions were assessed based on the calculated Cronbach's α coefficients, with values closer to 1 indicating better reliability. A Cronbach's α > 0.6 indicated acceptable reliability, while a value <0.6 suggested poor reliability (Tsai et al., 2017). In the development of the scale, previous studies (Blythe, 2017; Bender, 1978; Morrison, 1985; Hickey and Feldhacker, 2022; Kline, 2011; Wagner, 2015; Geerlinks, 2018; Feldhacker et al., 2022; Futagi et al., 2012; Bob et al., 2021; Futagi et al., 1992; Zafeiriou et al., 1995, 1999a,b; Zafeiriou, 2000; Konicarova and Bob, 2012, 2013; Blomberg, 2015; Zhao, 2019) have fully validated the theoretical framework of PRs and the dimensions of each type. Therefore, this study directly employed confirmatory factor analysis (CFA) to assess the model fit, without the need for exploratory factor analysis. CFA was conducted using Analysis of Moment Structure 28.0, with reference to the chi-square value/degrees of freedom (χ^2^/df), Root Mean Square Error of Approximation (RMSEA), Normed Fit Index (NFI), Tucker-Lewis Index (TLI), Incremental Fit Index (IFI), and Comparative Fit Index (CFI) to determine the model fit. Convergent and discriminant validity of the scale were reflected through factor loadings, composite reliability, average variance extracted (AVE), the square root of the AVE, and factor inter-correlations.

## 4 Results

### 4.1 Sample characteristics

A total of 555 students participated in this study. Of these, 234 students were involved in the pilot stage of scale development, including 102 boys and 132 girls, with an age range of 6 to 9 years and a mean age of 7.59 ± 0.71 years. In the formal stage of scale development, 321 students participated, comprising 171 boys and 150 girls, with an age range of 6 to 9 years and a mean age of 7.73 ± 0.71 years. The participants in both the pilot and formal stages showed no significant differences in terms of age, family background, education, or income levels.

### 4.2 Item analysis

After discussion by the expert panel, the initial draft scale, which consisted of 45 items across 13 dimensions, was refined to 37 items across 12 dimensions, resulting in the Primitive Reflex Integration Pilot Scale. The scale's framework includes the following reflexes: MR, ATNR, STNR, Tonic Labyrinthine Reflex (TLR), Spinal Galant Reflex (SGR), Spinal Perez Reflex (SPR), Landau Reflex (LR), Palmar Grasp Reflex (PAGR), Plantar Grasp Reflex (PLGR), Babkin Reflex (BNR), Hands Pulling Reflex (HPR), and Babinski Reflex (BIR). Based on the results of the IDM, CRM, and ICCM tests, a total of 20 items were initially identified for deletion. Specifically, 20 items were deleted according to the IDM test, 17 items based on the CRM test, and 7 items based on the ICCM test. After considering professional knowledge and ensuring content completeness, one item was adjusted and retained. Ultimately, 20 items were deleted, resulting in a preliminary version of the scale with 7 dimensions and 17 items (see Table 1).

**Table 1:** Results of the analysis of the preliminary version of the primitive reflex integration scale items.

**Dimension**	**Item**	**Item discrimination**	**Critical ratio**	**Cronbach's α coefficient**
TLR	1. Lower the head and look at the floor, then close the eyes. Observe if the body sways.	0.243	4.377	0.613
	2. Raise the head and look at the ceiling, then close the eyes. Observe if the body sways.	0.444	6.63	0.604
ATNR	1. During left turning, observe if the arms follow the head's movement and if there is any resistance in the head.^a^	0.508	4.591	0.626
	2. During right turning, observe if the arms follow the head's movement and if there is any resistance in the head.	0.317	3.506	0.618
SGR	1. When making a downward stroke 3 cm to the left of the spine, observe if the hip turns to the left.	0.360	3.630	0.595
	2. When making a downward stroke 3 cm to the right of the spine, observe if the hip turns to the right.	0.317	3.366	0.601
SPR	1. Observe if the head is lifted.	0.233	3.150	0.608
	2. Observe if the hip is lifted.	0.243	3.074	0.607
STNR	1. During support, observe if there is any locking of the elbow joint.	0.476	4.555	0.619
	2. During support, observe if there is any internal or external rotation of the arms.	0.254	3.096	0.604
	3. During support, observe if there is any finger flexion.	0.212	3.224	0.614
LR	1. Observe if there is any extension of both (or only one) of the legs.	0.677	6.594	0.602
	2. Observe if both (or one) of the feet are lifted.	0.667	6.486	0.604
MR	1. Observe if the movements are unsteady or lack fluidity.	0.550	7.151	0.592
	2. Observe if there is an inability to tighten both legs.	0.286	4.693	0.607
	3. Observe if both legs cannot be fully extended.	0.254	4.066	0.606
	4. Observe if both arms cannot be fully extended.	0.265	4.666	0.607

Significance level: *P* < 0.01; *P* < 0.001; Cronbach's α coefficient: Cronbach's α coefficient after Item deletion.

^a^Based on the comprehensiveness and content of each primitive reflex, a decision was made not to delete this item.

### 4.3 Reliability and validity analysis

**Figure 1:**
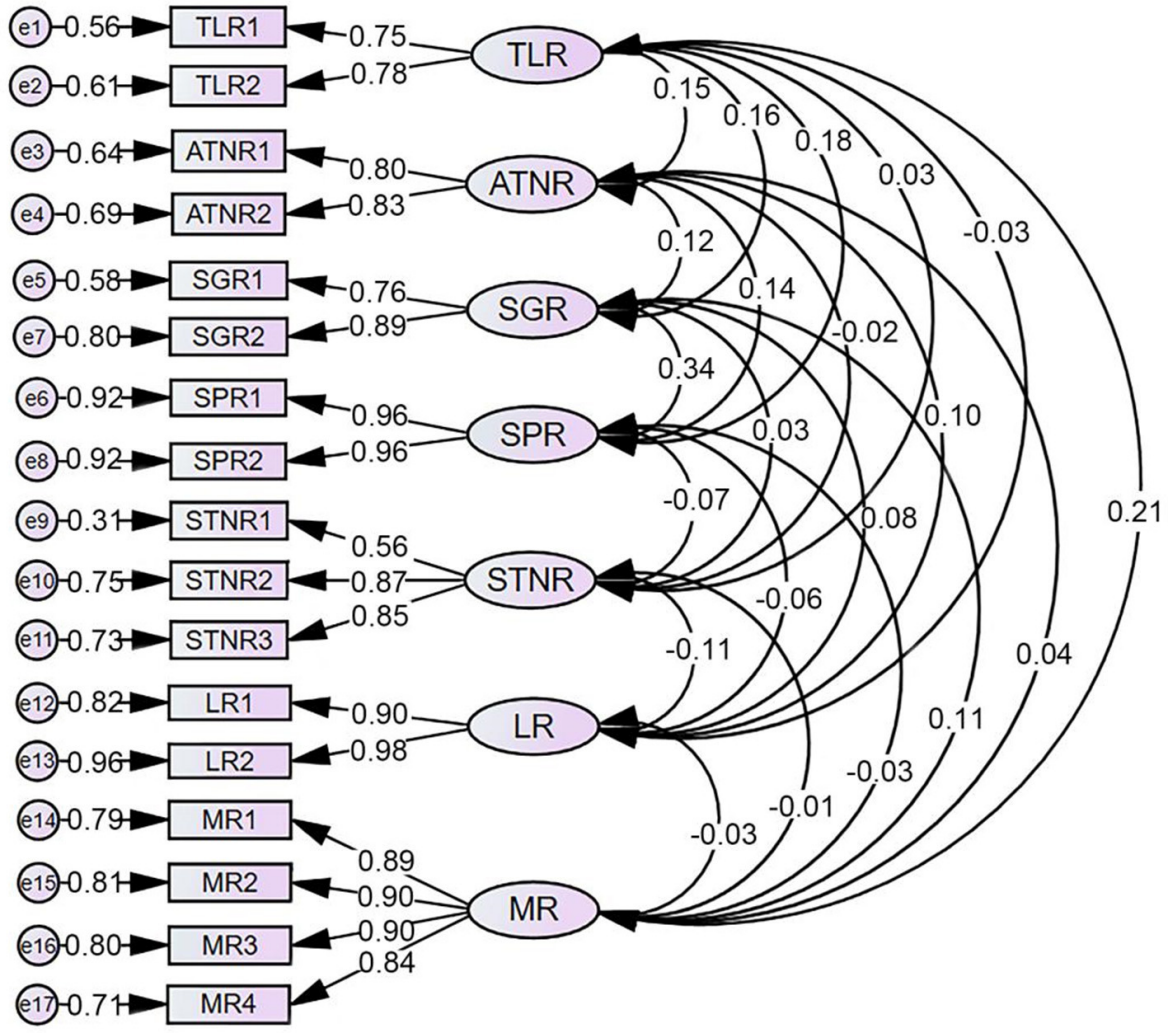
Model of the factor analysis results for the preliminary version of the primitive reflex integration measurement scale.

The Cronbach's α coefficients for the TLR, ATNR, SGR, SPR, STNR, LR, and MR dimensions were 0.730, 0.797, 0.808, 0.945, 0.767, 0.938, and 0.929, respectively, with values ranging from 0.730 to 0.945, all >0.7. Overall, the reliability was within an acceptable range, indicating good internal consistency of the scale. The model fit of the scale was further analyzed using the maximum likelihood method, with results showing [χ^2^/df = 1.631(<3) (Iacobucci, 2010), RMSEA = 0.044 (< 0.05) (Byrne, 2010), NFI = 0.950 (>0.9) (Lan et al., 2023), IFI = 0.932 (>0.9) (Ganotice et al., 2023), TLI = 0.972 (>0.9) (Heblich et al., 2023), and CFI = 0.980 (>0.9) (Iani et al., 2014)], indicating good model fit. The factor loadings between items and factors ranged from 0.56 to 0.98, all > 0.5 (Wei and Nguyen, 2020) (see Figure 1), indicating that the items corresponding to each latent variable have a certain level of representativeness; Composite reliability (CR) was >0.7 (Tobón and Luna-Nemecio, 2021); and the AVE for each factor was >0.5 (Fornell and Larcker, 1981; Yu et al., 2022), indicating that the convergent validity of each factor is ideal. Additionally, the square root of the AVE for each factor was greater than the correlation coefficients between factors, indicating good discriminant validity of the model (Wang et al., 2022) (see Table 2).

**Table 2:** Convergent and discriminant validity of the scale.

	**TLR**	**ATNR**	**SGR**	**SPR**	**STNR**	**LR**	**MR**
TLR	1.000						
ATNR	0.154	1.000					
SGR	0.163	0.120	1.000				
SPR	0.184	0.140	0.341	1.000			
STNR	0.031	−0.017	0.033	−0.070	1.000		
LR	−0.035	0.101	0.083	−0.062	−0.113	1.000	
MR	0.205	0.039	0.110	−0.033	−0.009	−0.028	1.000
CR	0.740	0.798	0.813	0.958	0.811	0.940	0.933
AVE	0.587	0.664	0.687	0.919	0.597	0.887	0.777
Square root of AVE	0.766	0.815	0.829	0.958	0.773	0.942	0.882

## 5 Discussion

This study ultimately developed the CPRIMS, a tool comprising 17 items across 7 dimensions, designed to scientifically assess the integration status of seven primitive reflexes in children. These reflexes include the TLR, ATNR, SGR, SPR, STNR, LR, and MR. By reviewing and analyzing existing research from both domestic and international sources, we summarized the formation, integration timeline, and symptoms of seven types of non-integration reflexes, providing a theoretical foundation for the scale's design.

ATNR typically begins to form *in utero*, usually appearing around 18 weeks of gestation, and gradually integrating within 3 to 9 months after birth (Berne, 2006; Blythe, 2002). Failure to integrate, in some cases, ATNR may impair children's ability to handle fine and gross motor tasks, leading to motor difficulties (Adams and Craft, 2014; Pecuch et al., 2020). STNR generally appears between 6 to 9 months of age, with integration typically completed within 9 to 11 months postnatally (Berne, 2006; Adams and Craft, 2014). Non-integrated STNR has been associated with poor organizational skills and abnormal hand-eye coordination (Wang et al., 2023). MR generally starts developing *in utero* around 9 to 12 weeks of gestation and integrates within 4 months after birth (Berne, 2006; Adams and Craft, 2014). Non-integrated MR is associated with emotional dysregulation, anxiety toward new activities, immature social behavior, and symptoms such as inattention or impulsivity (Adams and Craft, 2014; Futagi et al., 2012). TLR consists of two aspects: TLR forward and TLR backward. TLR forward typically forms around 12 weeks after birth, integrating within 3 to 4 months postnatally (Berne, 2006; Blythe, 2002). While TLR backward appears at birth and may take between 6 weeks and 3 years to integrate (Berne, 2006; Adams and Craft, 2014; Blythe, 2002; Pecuch et al., 2020). Non-integrated TLR can impact motor abilities, with TLR forward often associated with low muscle tone and TLR backward with high muscle tone, resulting in postural problems, balance difficulties, and even issues with visual and oculomotor control (Berne, 2006; Adams and Craft, 2014; Blythe, 2002). SGR typically begins developing *in utero* around 20 weeks of gestation and integrates within 9 months after birth (Adams and Craft, 2014). Non-integrated SGR may result in restlessness, affect short-term memory, disrupt time and spatial orientation development, and impact classroom attention (Adams and Craft, 2014). SPR usually appears at birth and integrates within 3 to 6 months postnatally (Wagner, 2015; Geerlinks, 2018). Non-integrated SPR can lead to hypersensitivity in the auditory and tactile systems, accompanied by sensory processing disorders, overactivity, and poor memory (Wagner, 2015). LR begins developing *in utero* around 4 weeks of gestation and takes about 3 years to fully integrate (Wagner, 2015; Geerlinks, 2018). Non-integrated LR can result in low muscle tone and potentially affect attention and vision (Wagner, 2015). Failure to integrate PRs can severely affect children's physical, emotional, and cognitive development, underscoring the critical importance of studying and monitoring reflex integration for healthy child development.

The results of this study indicate that CPRIMS achieves acceptable levels of internal consistency, reliability, and validity, demonstrating robust measurement performance. CPRIMS provides researchers with an objective and effective tool for scientifically evaluating primitive reflex integration in children. The development of this scale not only addresses gaps in existing measurement tools but also offers an effective method for identifying potential motor dysfunction, psychological issues, and neurodevelopmental disorders in children.

CPRIMS effectively overcomes the limitations of existing tools. First, the development of this scale enhances the adaptability of primitive reflex assessment tools to the evolving needs of modern society, because as time progresses and society changes, the characteristics and manifestations of unintegrated primitive reflexes may vary throughout the developmental process of children. Second, CPRIMS evaluates specific physical behaviors through direct observation and measurement, rather than assessing primitive reflex integration indirectly through comprehensive developmental information or other observable behaviors. This improves the feasibility and reliability of the assessment while minimizing bias in screening results. Third, CPRIMS follows a rigorous scale development process, incorporating clear validity and reliability indicators. This provides robust support for the credibility of the measurement method, enhancing its scientific rigor and applicability. Fourth, CPRIMS addresses the issue of inconsistent scoring standards by employing clear measurement procedures and standardized scoring systems to evaluate children's primitive reflex integration. This ensures consistency and reproducibility in the measurement and analysis of primitive reflexes, significantly enhancing its utility in both research and clinical contexts. Moreover, it lays a solid foundation for further studies exploring the relationship between developmental disorders and primitive reflexes. Notably, existing assessment tools lack clear age range specifications for their scales. Our scale is specifically developed for children aged 6 to 9 years, a stage that is crucial for both physical and psychological development. Evaluating primitive reflex integration during this period is particularly significant, as it provides valuable insights into children's developmental progress.

The results of this study indicate that CPRIMS achieves acceptable levels of internal consistency, reliability, and validity, demonstrating robust measurement performance. CPRIMS provides researchers with an objective and effective tool for scientifically evaluating primitive reflex integration in children. The development of this scale not only addresses gaps in existing measurement tools but also offers an effective method for identifying potential motor dysfunction, psychological issues, and neurodevelopmental disorders in children.

### 5.1 Limitations and prospects

This study has certain limitations. Firstly, as a preliminary investigation, the sample is restricted to three elementary schools in a city in China, which presents significant geographical limitations. This may impact the applicability of the scale in other regions and countries. Secondly, although the scale measures seven primitive reflexes closely related to children's physical and psychological development, there are many other important primitive reflexes. For example, the grasp reflex, which is difficult to observe in our sample due to its low elicitation rate, could not be included in the scale.

Future research could expand the survey regions and increase the sample size to facilitate more comprehensive analyses, thereby enriching the content and accuracy of the scale. This would further validate the scale's reliability and validity, and enable the establishment of normative data across diverse populations and age groups. These advancements would significantly enhance the scientific rigor and practical utility of the scale, supporting its broader adoption in both clinical and research applications.

## 6 Conclusions

CPRIMS is expected to become a powerful tool for assessing the integration status of PRs in children. It can help to identify early signs of developmental issues and provide crucial data support for personalized intervention plans. With further research and refinement, CPRIMS will see widespread use in practice, not only in clinical settings but also in educational and home interventions. Additionally, the widespread application of CPRIMS will promote a deeper understanding and prompt further research into neurodevelopmental disorders in children, further enhancing the efficacy of early diagnosis and intervention.

## Data Availability

The datasets presented in this study can be found in online repositories. The names of the repository/repositories and accession number(s) can be found in the article/Supplementary material.
