# Supplementary material for: Development of the children's primitive reflex integration assessment scale
Source: Front Psychol. 2025 Jan 22;16:1495990. doi: 10.3389/fpsyg.2025.1495990 (PMC11794803; doi:10.3389/fpsyg.2025.1495990)
Supplement: Supplementary file 1 [file Data_Sheet_1.docx]

**Appendix 1:**

**Process Table for Developing the Pilot Test Scale**

| **Primitive Reflex** | **Literature Source** | | **Clinical Experience** | **Final Decision** | |
| --- | --- | --- | --- | --- | --- |
| MR | 1) Participants stand with feet together, arms bent at a 45-degree angle, wrists bent. The researcher stands behind and quickly tilts the participant's head backward, triggering the MR.  2) Participants lie supine, head suddenly drops, causing the head to extend, upper limbs to abduct, then adduct and flex, triggering the MR. | | Participants sit, try to bring legs close to the chest, hug knees tightly, then lie down with limbs extended as much as possible. | Participants need to complete the hugging motion 1-2 times (start sitting, feet off the ground, legs bent, arms hugging the legs, legs as close to the chest as possible, and finally lying down slowly, fully extending arms and legs). | |
| ATNR | 1) Participants stand with feet together, arms straight and level with shoulders, hands relaxed at wrists, triggering the ATNR.  2) Participants extend their arms in front, arms remain in the same position while the researcher moves the participant's head, triggering the ATNR. | | Participants stand with feet together, arms extended forward, eyes closed, researcher rotates participant's head left and right. | Participants stand, feet together, arms extended forward, eyes closed, instructed to relax while researcher rotates participant's head left and right. | |
| STNR | 1) Participants in a quadruped position. Instructed to look at knees for 5 seconds, then at the ceiling for 5 seconds, triggering the STNR.  2) Participants in a quadruped position, slowly move head downwards as if looking between thighs. Hold for up to 5 seconds, then slowly move head upwards as if looking at the ceiling, repeat up to 6 times, triggering the STNR. | | 1) Participants lie on the ground, hands and knees on the ground.  2) Participants place hands flat on the ground, fingers pointing forward, elbows slightly bent, scapulae extended, relaxed, back straight, thoracic spine straight, lumbar spine slightly arched, then slowly bend the head backward. | 1) Participants in a quadruped position.  2) Participants in a quadruped position, complete cat pose (exhale while lowering head and arching back, inhale while lifting head and arching the back). | |
| TLR | TLR forwards | Participants stand with feet together, arms straight at sides, move head up/down, close eyes, hold for 10 seconds.  TLR forward: Looking at the ceiling triggers TLR.  TLR backward: Looking at feet triggers TLR. | Participants stand with feet together on a soft surface, head bent forward, eyes open, head bent forward, eyes closed; head bent backward, eyes open, head bent backward, eyes closed. | | TLR forwards: Participants stand, feet together, looking ahead, lower head, look at the floor, close eyes, hold for 10 seconds; TLR backward: Participants stand, feet together, look at the ceiling, close eyes, hold for 10 seconds. |
|  | TLR backwards |  |  |  |  |
| SGR | Participants in a quadruped position, researcher brushes down the back from shoulder to lumbar spine (no more than 2 times); complete on one side, then the other side, triggering the SGR. | | Participants lie prone or with limbs touching the ground, use a fingernail or pen tip to draw 10 cm above and below the waist, 3 cm lateral to the spine. | | Participants in a quadruped position, knees hip-width apart, eyes level with the ground, use a brush to draw from 10 cm above to 10 cm below the first lumbar vertebra, 3-4 cm lateral to the spine. |
| SPR | Participants lie prone, researcher uses a brush to sweep from the coccyx to the top of the spine, triggering the SPR. | | Participants lie prone, use a finger to sweep from the coccyx to the neck. | | Participants lie prone, use a brush along the spine from the coccyx to the top of the spine. |
| LR | Participants lift head and chest while lying prone. | | Participants lie prone, arms extend backward, lift head and chest. | | Participants lie prone, arms at sides, instructed to lift head and chest as much as possible. |
| PAGR | 1) Researcher places an index finger in the infant's palm, triggering the PAGR.  2) Researcher presses on the infant's palm, triggering the PAGR. | | Participants lie supine, place a pen or finger into the participant's palm. | | Participants lie supine, use a pen or finger to press into the palm from the ulnar side (little finger side). |
| PLGR | Researcher presses thumb on the sole of the foot behind the toes, triggering the PLGR. | | Participant's sole receives pressure, triggering the PLGR. | | Participants lie supine, use a pen or finger to press on the participant's forefoot arch. |
| BNR | Participants lie supine, researcher presses on the participant's palm, triggering the BNR. | | Participants lie supine, researcher presses on the participant's palm. | Participants lie supine, hands at sides, instructed to slightly open mouth, researcher presses on the participant's palm. | |
| HPR | Researcher holds the infant's forearm near the wrist and pulls toward a sitting position, triggering the HPR. | | Participants lie supine, forearms bent, researcher places thumbs in the participant's palms and holds the wrist, pulling in different directions. | Participants lie supine, forearms bent, researcher places thumbs in the participant's palms, other fingers grasp the wrist, pulling upwards, downwards, inward, and outward. | |
| BIR | 1) Use a brush to stroke the outer side of the sole from heel to big toe, triggering the BIR.  2) Researcher strokes the outer side of the participant's sole from heel to the head of the fifth metatarsal, triggering the BIR. | | Use a pen tip to stroke the outer side of the sole from the heel upwards then inwards. | Participants lie supine, use a brush to stroke the outer side of the sole from heel to big toe. | |

**Appendix 2:**

**Focus Group Discussion**

Interviews with special education teachers (N=10) and pediatricians (N=2) were conducted using both open-ended and closed-ended questions to develop the Primitive Reflex Integration Assessment Scale.

| **Closed-ended Questions** | **Open-ended Questions** |
| --- | --- |
| - Interview Date: - Interview Location: - Name: - Gender: - Age: - Educational Background: - Title: - Teaching Experience: | - Based on your clinical experience and understanding of the primitive reflex integration level of children in grades 1-2 (ages approximately 6-9), we have set up 12 dimensions reflecting the integration status of primitive reflexes in children aged 6-9. These dimensions include the Moro Reflex（MR）, Asymmetric Tonic Neck Reflex (ATNR), Symmetric Tonic Neck Reflex (STNR), Tonic Labyrinthine Reflex (TLR), Spinal Galant Reflex (SGR), Spinal Perez Reflex (SPR), Landau Reflex (LR), Palmar Grasp Reflex（PAGR）, Plantar Grasp Reflex（PLGR）, Babkin Reflex（BNR）, Hands Pulling Reflex（HPR）, and Babinski Reflex（BIR）. Do you think this is reasonable? Are there any dimensions that should be added or removed? - Do you think the content of the 12 dimensions for primitive reflex integration is reasonable? Are there any additions or deletions needed? - Do you think the content of the evaluation items within the 12 dimensions is reasonable? Are there any additions or deletions needed? |

**Appendix 3:**

**Primitive Reflex Integration Assessment Scale Item Bank**

**Specially Appointed Professor:**

Below is the item bank for the "Primitive Reflex Integration Assessment Scale." Please check the most appropriate option for each item and mark "√" in the degree code 1, 2, 3, 4, 5. 1 means "very inappropriate," 5 means "very appropriate." Each suggestion you provide is very important to this research. Thank you for your careful guidance and support!

| Dimension | Very Inappropriate | Inappropriate | Unclear | Appropriate | Very Appropriate | Comments |
| --- | --- | --- | --- | --- | --- | --- |
| Tonic Labyrinthine Reflex (forward) | 1 | 2 | 3 | 4 | 5 |  |
| Tonic Labyrinthine Reflex (backward) | 1 | 2 | 3 | 4 | 5 |  |
| Asymmetric Tonic Neck Reflex | 1 | 2 | 3 | 4 | 5 |  |
| Spinal Galant Reflex | 1 | 2 | 3 | 4 | 5 |  |
| Spinal Perez Reflex | 1 | 2 | 3 | 4 | 5 |  |
| Symmetric Tonic Neck Reflex | 1 | 2 | 3 | 4 | 5 |  |
| Landau Reflex | 1 | 2 | 3 | 4 | 5 |  |
| Moro Reflex | 1 | 2 | 3 | 4 | 5 |  |
| Palmar Grasp Reflex | 1 | 2 | 3 | 4 | 5 |  |
| Babkin Reflex | 1 | 2 | 3 | 4 | 5 |  |
| Hands Pulling Reflex | 1 | 2 | 3 | 4 | 5 |  |
| Plantar Grasp Reflex | 1 | 2 | 3 | 4 | 5 |  |
| Babinski Reflex | 1 | 2 | 3 | 4 | 5 |  |

**Appendix 4:**

**Primitive Reflex Integration Assessment Scale Item Bank**

**Specially Appointed Professor:**

Below is the item bank for the "Primitive Reflex Integration Assessment Scale." Please check the most appropriate option for each item and mark "√" in the degree code 1, 2, 3, 4, 5. 1 means "very inappropriate," 5 means "very appropriate." Each suggestion you provide is very important to this research. Thank you for your careful guidance and support!

| **Item** | Very Inappropriate | Inappropriate | Unclear | Appropriate | Very Appropriate | Comments |
| --- | --- | --- | --- | --- | --- | --- |
| Participants stand with feet together, looking forward, lower head, look at the floor, then close eyes. | | | | | | |
| 1. Does the body sway or become unstable? | 1 | 2 | 3 | 4 | 5 |  |
| 2. Does the participant hold their breath? | 1 | 2 | 3 | 4 | 5 |  |
| 3. Does muscle tension occur? | 1 | 2 | 3 | 4 | 5 |  |
| Participants stand with feet together, look at the ceiling, then close eyes. | | | | | | |
| 4. Does the body sway or become unstable? | 1 | 2 | 3 | 4 | 5 |  |
| 5. Does the participant hold their breath? | 1 | 2 | 3 | 4 | 5 |  |
| 6. Does muscle tension occur? | 1 | 2 | 3 | 4 | 5 |  |
| Participants stand with feet together, arms extended forward, eyes closed, instructed to relax, researcher rotates participant's head to the left and right. | | | | | | |
| 7. Does the arm follow the head during left rotation? | 1 | 2 | 3 | 4 | 5 |  |
| 8. Does the arm follow the head during right rotation? | 1 | 2 | 3 | 4 | 5 |  |
| 9. Is there resistance in the head during left rotation? | 1 | 2 | 3 | 4 | 5 |  |
| 10. Is there resistance in the head during right rotation? |  |  |  |  |  |  |
| Participants in a quadruped position, knees hip-width apart, eyes level with the ground, researcher uses a brush to stroke 3-4 cm lateral to the spine at the level of the first lumbar vertebra, 10 cm above and below. | | | | | | |
| 11. When stroking 3 cm to the left of the spine downward, does the hip turn to the left? | 1 | 2 | 3 | 4 | 5 |  |
| 12.When stroking 3 cm to the right of the spine downward, does the hip turn to the right? | 1 | 2 | 3 | 4 | 5 |  |
| Participants lie prone, researcher uses a brush to stroke along the spine from the coccyx to the top. | | | | | | |
| 13. Does the thoracic spine bend downward? | 1 | 2 | 3 | 4 | 5 |  |
| 14. Does the head lift? | 1 | 2 | 3 | 4 | 5 |  |
| 15. Does the hip lift? | 1 | 2 | 3 | 4 | 5 |  |
| 16. Do the arms bend? | 1 | 2 | 3 | 4 | 5 |  |
| 17. Do the legs bend? | 1 | 2 | 3 | 4 | 5 |  |
| Participants in a quadruped position, completing the cat pose (exhale while lowering the head and arching the back, inhale while lifting the head and arching the back). | | | | | | |
| 18. Do the elbows lock during support? | 1 | 2 | 3 | 4 | 5 |  |
| 19. Do the hands rotate inward or outward during support? | 1 | 2 | 3 | 4 | 5 |  |
| 20. Do the scapulae retract during support? | 1 | 2 | 3 | 4 | 5 |  |
| 21. Do the fingers bend during support? | 1 | 2 | 3 | 4 | 5 |  |
| 22. Does the back arch upward during support? | 1 | 2 | 3 | 4 | 5 |  |
| 23. Does the hip move backward during the cat pose while lowering the head? | 1 | 2 | 3 | 4 | 5 |  |
| Participants lie prone, arms at sides, instructed to lift the head and chest as much as possible. | | | | | | |
| 24. Is the head unable to lift? | 1 | 2 | 3 | 4 | 5 |  |
| 25. Is the chest unable to lift? | 1 | 2 | 3 | 4 | 5 |  |
| 26. Do the legs extend? | 1 | 2 | 3 | 4 | 5 |  |
| 27. Do the feet lift? | 1 | 2 | 3 | 4 | 5 |  |
| Participants need to complete the hugging motion 1-2 times (starting from a sitting position, feet off the ground, legs bent, arms hugging the legs, legs as close to the chest as possible, and finally lying down slowly, fully extending the arms and legs). | | | | | | |
| 28. Is the motion not smooth? | 1 | 2 | 3 | 4 | 5 |  |
| 29. Are the legs unable to tighten? | 1 | 2 | 3 | 4 | 5 |  |
| 30. Are the legs unable to fully extend? | 1 | 2 | 3 | 4 | 5 |  |
| 31. Are the arms unable to fully extend? | 1 | 2 | 3 | 4 | 5 |  |
| 32. In the supine position, head tilts 30° backward from the initial position, then returns to the initial position, is there dizziness? | 1 | 2 | 3 | 4 | 5 |  |
| 33. In the supine position, with hips and knees flexed, legs quickly pulled out, is there muscle tension in the lower limbs? | 1 | 2 | 3 | 4 | 5 |  |
| 34. In the supine position, when tapping the ground 10-15 cm from the ear, is there a response? | 1 | 2 | 3 | 4 | 5 |  |
| 35. In the supine position, with hands placed on the chest, lightly tap both upper arms, is there a response? | 1 | 2 | 3 | 4 | 5 |  |
| 36. In the supine position, when waving a hand in front of the participant's eyes (>8 cm), is there a response? | 1 | 2 | 3 | 4 | 5 |  |
| Participants lie supine, use a pen or finger to press into the palm from the ulnar side (little finger side). | | | | | | |
| 37. Do the fingers bend or make a fist? | 1 | 2 | 3 | 4 | 5 |  |
| Participants lie supine, hands at sides, instructed to slightly open mouth, researcher presses on the participant's palm. | | | | | | |
| 38. Does the mouth open wider? | 1 | 2 | 3 | 4 | 5 |  |
| 39. Do the muscles in the jaw or neck contract? | 1 | 2 | 3 | 4 | 5 |  |
| Participants lie supine, forearms bent, researcher places thumbs in the participant's palms, other fingers grasp the wrist, pulling upwards, downwards, inward, and outward. | | | | | | |
| 40. Is there a change in arm tension when pulling upwards? | 1 | 2 | 3 | 4 | 5 |  |
| 41. Is there a change in arm tension when pulling downwards? | 1 | 2 | 3 | 4 | 5 |  |
| 42. Is there a change in arm tension when pulling inward? | 1 | 2 | 3 | 4 | 5 |  |
| 43. Is there a change in arm tension when pulling outward? | 1 | 2 | 3 | 4 | 5 |  |
| Participants lie supine, researcher presses thumb between the participant's toes and arch. | | | | | | |
| 44. Do the toes bend? | 1 | 2 | 3 | 4 | 5 |  |
| Participants lie supine, use a brush to stroke the outer side of the sole from heel to big toe. | | | | | | |
| 45. Does the big toe extend (bend towards the dorsum), and the other four toes fan out? | 1 | 2 | 3 | 4 | 5 |  |

**Appendix 5:**

**Pre-test Scale**

| Dimension | Item | Not Present | Slight | **Noticeable** | **Very Noticeable** |
| --- | --- | --- | --- | --- | --- |
| Tonic Labyrinthine Reflex | Participants stand with feet together. | | | | |
|  | 1. Lower head, look at the floor, then close eyes, hold for 10 seconds, does the body sway? |  |  |  |  |
|  | 2. Look at the ceiling, then close eyes, hold for 10 seconds, does the body sway? |  |  |  |  |
| Asymmetric Tonic Neck Reflex | Participants stand with feet together, arms extended forward, eyes closed, instructed to relax, researcher rotates participant's head to the left and right. | | | | |
|  | 3. Does the arm follow the head during left rotation, is there resistance in the head? |  |  |  |  |
|  | 4. Does the arm follow the head during right rotation, is there resistance in the head? |  |  |  |  |
| Spinal Galant Reflex | participants in a quadruped position, knees hip-width apart, eyes level with the ground, researcher uses a brush to stroke 3-4 cm lateral to the spine at the level of the first lumbar vertebra, 10 cm above and below. | | | | |
|  | 5. Does the hip rotate to the left when stroking the left side? |  |  |  |  |
|  | 6. Does the hip rotate to the right when stroking the right side? |  |  |  |  |
| Spinal Perez Reflex | Participants lie prone, researcher uses a brush to stroke along the spine from the coccyx to the top. | | | | |
|  | 7. Does the thoracic spine bend downward? |  |  |  |  |
|  | 8. Does the head lift? |  |  |  |  |
|  | 9. Does the hip lift? |  |  |  |  |
|  | 10. Do the arms and legs bend? |  |  |  |  |
| Symmetric Tonic Neck Reflex | Participants in a quadruped position, completing the cat pose (exhale while lowering the head and arching the back, inhale while lifting the head and arching the back). | | | | |
|  | 11. Do the elbows lock during support? |  |  |  |  |
|  | 12. Do the arms rotate inward or outward during support? |  |  |  |  |
|  | 13. Do the scapulae retract during support? |  |  |  |  |
|  | 14. Do the fingers bend during support? |  |  |  |  |
|  | 15. Does the back arch upward during support? |  |  |  |  |
|  | 16. Does the hip move backward during the cat pose while lowering the head? |  |  |  |  |
| Landau Reflex | Participants lie prone, arms at sides, instructed to lift the head and chest as much as possible. | | | | |
|  | 17. Are the head and chest unable to lift? |  |  |  |  |
|  | 18. Do the legs (single leg) extend? |  |  |  |  |
|  | 19. Do the feet (single leg) lift? |  |  |  |  |
| Moro Reflex | Participants need to complete the hugging motion 1-2 times (starting from a sitting position, feet off the ground, legs bent, arms hugging the legs, legs as close to the chest as possible, and finally lying down slowly, fully extending the arms and legs). | | | | |
|  | 20. Is the motion not smooth? |  |  |  |  |
|  | 21. Are the legs unable to tighten? |  |  |  |  |
|  | 22. Are the legs unable to fully extend? |  |  |  |  |
|  | 23. Are the arms unable to fully extend? |  |  |  |  |
|  | 24. In the supine position, head tilts 30° backward from the initial position, then returns to the initial position, is there dizziness? | | | | |
|  | 25. In the supine position, with hips and knees flexed, legs quickly pulled out, is there muscle tension in the lower limbs? |  |  |  |  |
|  | 26. In the supine position, when tapping the ground 10-15 cm from the ear, is there a response? |  |  |  |  |
|  | 27. In the supine position, with hands placed on the chest, lightly tap both upper arms, is there a response? |  |  |  |  |
|  | 28. In the supine position, when waving a hand in front of the participant's eyes (>8 cm), is there a response? |  |  |  |  |
| Palmar Grasp Reflex | Participants lie supine, use a pen or finger to press into the palm from the ulnar side (little finger side). | | | | |
|  | 29. Do the fingers bend or make a fist? |  |  |  |  |
| Babkin Reflex | Participants lie supine, hands at sides, instructed to slightly open mouth, researcher presses on the participant's palm. | | | | |
|  | 30. Does the mouth open wider? |  |  |  |  |
|  | 31. Do the muscles in the jaw or neck contract? |  |  |  |  |
| Hands Pulling Reflex | Participants lie supine, forearms bent, researcher places thumbs in the participant's palms, other fingers grasp the wrist, pulling upwards, downwards, inward, and outward. | | | | |
|  | 32. Is there a change in arm tension when pulling upwards? |  |  |  |  |
|  | 33. Is there a change in arm tension when pulling downwards? |  |  |  |  |
|  | 34. Is there a change in arm tension when pulling inward? |  |  |  |  |
|  | 35. Is there a change in arm tension when pulling outward? |  |  |  |  |
| Plantar Grasp Reflex | Participants lie supine, researcher presses thumb between the participant's toes and arch. | | | | |
|  | 36. Do the toes bend? |  |  |  |  |
| Babinski Reflex | Participants lie supine, use a brush to stroke the outer side of the sole from heel to big toe. | | | | |
|  | 37. Does the big toe extend (bend towards the dorsum), and the other four toes fan out? |  |  |  |  |

**Appendix 6:**

**Preliminary Version of the Scale**

| Dimension | Item | Not Present | Slight | **Noticeable** | **Very Noticeable** |
| --- | --- | --- | --- | --- | --- |
| Tonic Labyrinthine Reflex | Participants stand with feet together. | | | | |
|  | 1. Lower head, look at the floor, then close eyes, hold for 10 seconds, does the body sway? |  |  |  |  |
|  | 2. Look at the ceiling, then close eyes, hold for 10 seconds, does the body sway? |  |  |  |  |
| Asymmetric Tonic Neck Reflex | Participants stand with feet together, arms extended forward, eyes closed, instructed to relax, researcher rotates participant's head to the left and right. | | | | |
|  | 3. Does the arm follow the head during left rotation, is there resistance in the head? |  |  |  |  |
|  | 4. Does the arm follow the head during right rotation, is there resistance in the head? |  |  |  |  |
| Spinal Galant Reflex | Participants in a quadruped position, knees hip-width apart, eyes level with the ground, researcher uses a brush to stroke 3-4 cm lateral to the spine at the level of the first lumbar vertebra, 10 cm above and below. | | | | |
|  | 5. Does the hip rotate to the left when stroking the left side? |  |  |  |  |
|  | 6. Does the hip rotate to the right when stroking the right side? |  |  |  |  |
| Spinal Perez Reflex | Participants lie prone, researcher uses a brush to stroke along the spine from the coccyx to the top. | | | | |
|  | 7. Does the head lift? |  |  |  |  |
|  | 8. Does the hip lift? |  |  |  |  |
| Symmetric Tonic Neck Reflex | Participants in a quadruped position, completing the cat pose (exhale while lowering the head and arching the back, inhale while lifting the head and arching the back). | | | | |
|  | 9. Do the elbows lock during support? |  |  |  |  |
|  | 10. Do the arms rotate inward or outward during support? |  |  |  |  |
|  | 11. Do the fingers bend during support? |  |  |  |  |
| Landau Reflex | Participants lie prone, arms at sides, instructed to lift the head and chest as much as possible. | | | | |
|  | 12. Do the legs (single leg) extend? |  |  |  |  |
|  | 13. Do the feet (single leg) lift? |  |  |  |  |
| Moro Reflex | Participants need to complete the hugging motion 1-2 times (starting from a sitting position, feet off the ground, legs bent, arms hugging the legs, legs as close to the chest as possible, and finally lying down slowly, fully extending the arms and legs). | | | | |
|  | 14. Is the motion not smooth? |  |  |  |  |
|  | 15. Are the legs unable to tighten? |  |  |  |  |
|  | 16. Are the legs unable to fully extend? |  |  |  |  |
|  | 17. Are the arms unable to fully extend? |  |  |  |  |

**Supplementary Material:**

**Criteria for Judging the Degree of Primitive Reflex Integration:**

The degree of primitive reflex integration in children is classified as mild, moderate, or severe based on the item scores.

- For dimensions with 2 items, the total score: 1-2 points is mild; 3-4 points is moderate; 5-6 points is severe.
- For dimensions with 3 items, the total score: 1-3 points is mild; 4-6 points is moderate; 7-9 points is severe.
- For dimensions with 4 items, the total score: 1-4 points is mild; 5-8 points is moderate; 9-12 points is severe.
